# Moving On? The Act of Remembering Humanitarian Aid and the Shaping of Normative Experiences

**DOI:** 10.1163/2208522x-bja10065

**Published:** 2024-12-27

**Authors:** Bertrand Taithe

**Affiliations:** Humanitarian and Conflict Response Institute, University of Manchester, Manchester, UK

**Keywords:** learning, experience, affective communities, history, memory, humanitarian aid

## Abstract

This essay draws on the experience of ‘get-togethers’ among veteran field workers recorded in 2016 who recalled work experience from the 1980s–often stressing in their account their memories and the part this period played in their lives. This source can now help us analyse the formation of meaningful experience of the concept of experience. Using further archives of normative processes which drew from ‘lessons learnt’ and ‘experience’ to shape the rulebooks of the 1990s’ humanitarian aid industry, and using interviews and oral history as well as new archives and diaries, this essay will consider how humanitarians have reflected on normative experiences. The first section of the essay will be devoted to the experiences arising from work in the great refugee camps of the 1980s, where many humanitarians developed their skills. The second section develops from this and considers how camps became the site of norm-setting for many actors and organisations of the humanitarian sector in the 1990s. The third section of the article reflects on the legitimacy arising from field work and humanitarian experience, focusing on the sociability of experience and how this cultural capital was then instrumentalised. The final section ends on the itinerant humanitarian exhibition which was deployed at the end of the presence (2020–21) of one of the largest medical NGOs in Cambodia. Through this Médecins sans Frontières exhibition the essay will reflect on how experience and legitimacy were constructed and finally historicised and memorialized.

This essay reflects on the experience of being a ‘veteran’ humanitarian.^1^ The category of veteran for a civilian form of life-altering volunteering is obviously not constructed by structures such as the British Legion or veteran support groups. Yet, since the 2010s at least, ‘get-togethers’ and networks have focused on establishing affective communities grounding past experiences in an emotional memorial process which reinforces the cohesion of an international professional group.^2^ This memorial process has taken more institutional forms too, and the UN ‘Humanitarian Day’ every 19 August is an occasion for formal celebrations of dead humanitarians.^3^ Throughout the world monuments, for instance in Canada, Britain and Australia, have also reinforced this sense that humanitarians have, like the military before them, a shared identity forged in a common experience of mourning lost ones and which invites memorial rituals.^4^

Private commemorations and veteran groups predate the monuments and the formal effort to establish a narrative of humanitarian endeavours in which emotions – joy, nostalgia, sadness and indignation – provide a common register of expression. These emotions help give structure to complex professional identities, often notable by their career discontinuities or brief intensity.^5^ The get-togethers events act on a desire to recall key moments of individuals’ past, but they also frame the emotional work required by ‘trauma communities’ as defined by Peter Leese.^6^ They are, of course, also imbued with some nostalgia and the pleasures of a narcissistic return to the heydays of humanitarians’ performances of their ‘moral labour’. To use Anne-Meike Fechter’s definition, this is a specific notion of labour defined more by its performance than by its effectiveness, which implies that humanitarian work affects at an existential level those who undertake it, even if they fail to deliver fully the aid they intended to give.^7^ When veterans meet, years after the events and when many have long moved on from humanitarian labour, these group gatherings open up the possibility of engaging with the emotional depth of their experience.^8^ Some sought a kind of ‘closure’, while others reconnected disparate moments of their lives to what had become a foundational moment of a complex career. By looking in particular at veterans of a key phase of contemporary humanitarian aid in the 1980s, one can witness and analyse the emotional loading of reform initiatives and careers which referred to this period.

This essay draws on participant observations as well as the experience of setting up a humanitarian archive collecting the records and documents pertaining to the working lives of humanitarians. Additional work on creating synchronous records of humanitarian operations while observing them also informs this approach.^9^ Yet it is the experience of ‘get-togethers’ among veteran field workers in 2015 which began in earnest this research.^10^ At this meeting a range of humanitarian workers and some of the people they worked for and with recalled the refugee camp experience from the 1980s. This period corresponds to the proliferation of non-governmental organisations during what Kevin O’Sullivan calls the NGO moment, when large international humanitarian organisations emerged or redefined their remit while growing considerably in strength and financial acumen.^11^

The focus of the event that took place in and around the town of Aranyaprathet in north-eastern Thailand was to discuss life encounters and daily experiences and walk through the sites where camps had once existed.^12^ I was allowed in the gathering as a historian who sought to gather some oral history and capture the voices and recollections of humanitarians to compensate for the loss of physical archives. My small field recording equipment captured the joyful, tearful, occasionally indignant and sometimes solemn brouhaha of the small crowd of veterans and refugees. Socialising experiences and, therefore, constructing them, was the central transaction among a diverse group comprising many different periods of a long humanitarian intervention (1979–92). The refugee camps at the Thai border of Cambodia were improvised in great haste in October 1979. Very quickly they had become large cities and were among the largest humanitarian interventions of the era and a much-publicised example of coordinated humanitarian rule over a displaced people.^13^

The people attending the meeting were of various origins (see fig. 1). Some from Cambodia, some from the UN world, many Americans, Irish and British, Europeans (including French), Indians and Thais. Some knew each other, many were alone but knew someone known by another. A web of emotional bonds was constantly reinvented. Ratha Touch, a Cambodian refugee who had become a humanitarian worker in the camps before migrating to the USA, thus insisted on making connections through the Médecins sans Frontières (MSF) workers I knew. The sharing of intimate experience arose from more than just belonging in the crowd; it fed from the recalling enabled by the megaphone which went from one person to the next in no particular order.

They were partaking in the remembering of the largest humanitarian responses of the post – World War era. Over fourteen years, thousands rubbed shoulders then, but the ones who made the journey only met thirty years later. They had memories to share of their experience. Yet this effort of recollection, this essay argues, has to be read as something more profound and significant than a narcissistic holiday among lost friends.

Memorialisation is not simply a nostalgic exercise reflecting on how humanitarians lived their work in the 1980s; it is primarily a process of reflecting on their experience as a normative and formative education which later constituted the ‘humanitarian system’.^14^ The affective community of ‘veteran humanitarians’ became epistemic communities, able to translate knowledge into some power of norms and standards of humanitarian work over the ensuing decades.^15^ Those humanitarians who carried on working beyond their first experience shaped what humanitarian work ought to be. Their records show how they constituted informal and increasingly rationalised collections of materials, which drew from ‘lessons learnt’ and ‘experience’ to shape the earlier rulebooks of the 1990s humanitarian aid industry.^16^ The reformist drive of this period was led by individuals like British humanitarians John Borton, Peter Walker and others, and it amounted to an effort to build on experience and develop a knowledge base for a reform of practice akin to the recent improvements in airline safety.^17^ This led to new normative efforts, allegedly conceived on a mountaintop around a six-pack of beer,^18^ named the Sphere project, which joined a wider range of ‘professionalising initiatives’.^19^

The central preoccupation of this essay will thus be to show how the recognition of experience and legitimacy drew from emotional memories of humanitarian pasts. It will show how cultural practices enabled them, as part of a cognitive and affective process of belonging and shaping the humanitarian endeavour. Where reforming humanitarianism may appear to be uncontestably needed and imbued with a fundamental rationale to do not just good, but better, the humanitarian memorial processes underpinning this reformist agenda were more fragile than they may seem. The ‘moral labour’ of aid workers, as an activity whose ‘outcome is achieved in its performance’, was also re-enacted over time through the emotionally charged memorial process of storytelling to a peer group and to the communities in which humanitarians worked.^20^ The final examples supporting this analysis will be from an itinerant humanitarian exhibition, ‘Connecting Hands’, which travelled through Cambodia in autumn 2019 and winter 2020 with the view to bring together humanitarian workers and evoke the history of a sequence of humanitarian ‘missions’ in the country (see fig. 2) and a T-shirt exhibition which took place in Paris in spring 2022 (see fig. 3).^21^ The exhibition in Cambodia embraced over forty years of history in the country and brought about moving encounters, yet it also signalled the end of a prolonged humanitarian engagement. Arguably it illustrated how the experience of humanitarian work differed and how humanitarian workers could experience labours of aid in a very contrasted manner. This essay adds thus to a growing literature devoted to the history of humanitarian work as emotional and moral labour,^22^ fuelled by contradictions and dilemmas but nevertheless worthy of historical interpretation.^23^

I was present at both events book ending this article. The first was a ‘gettogether’ of a group which, taken prosopographically as a collective autobiography, represented a significant epistemic community engaging in a self-defining act of recollection. The second sought to establish an authoritative memento and timeline of the different types of missions that MSF had led over the years in Cambodia from the Cold War border camps era to the deployment of antiretroviral treatments for hepatitis C.^24^ The former belonged to a specific act of remembrance and was a celebratory act which also entailed commemorative processes for Cambodians who had lived in the camps and found life back in Cambodia difficult. The latter also invited participatory engagement from Cambodians whose professional experiences and lives had been shaped by encounters with a large medical humanitarian organisation.^25^ In a crucial sense the experience of being a humanitarian or a humanitarian employee diverged and diverge still. Similarly, the experience of being a humanitarian at the borders was for many a one-off experience – albeit one that shaped the course of their lives – while for others it became a normative training which later inspired policy reforms.

## 1 Remembering the Camps

The recollections of the camps focused on good-willed interventions at a time when hundreds of thousands of Cambodians crossed the minefields at the border of Thailand to seek refuge and flee the Vietnamese invasion of 1979. By October 1979 an archipelago of camps had settled on the borders of Thailand, only one of which, Khao I Dang, was fully in Thai territory and managed by the United Nations High Commissioner for Refugees (UNHCR). By 1982 all the border camps were run by a bespoke unitary agency, the United Nations Border Relief Operation (UNBRO), which coalesced the over 120 international NGOs working in the area.^26^ Each camp had a political and social identity and, after curfew, all experienced violence and trafficking. ‘There were a lot of things going on. There was a black market, people getting shot on the way.’^27^

Better resourced than any of the other mass refugee situations at the time, the huge Cambodian camps were transient structures, built of bamboo and straw. The camps were tolerated and contained by the Thai authorities at their border, but the Thai government had not signed up to the 1951 refugee convention or its amendments, and only subscribed partially to international laws.^28^ Ultimately, at the time of the mass repatriation of refugees between 1991 and 1993, these camps were burned down by Thai authorities keen to prevent the long-term settlement of a large Cambodian population.^29^ The page was closed and Thailand and Cambodia returned to their long history of border bickering. So, when we all gathered by the border in January 2015, there was then nothing to see. The little concrete of the foundations of a few buildings had been pulverised. The site of the UNHCR camp, Khao I Dang, was an arboretum planted under royal authority to restore the site to a state of nature. The largest complex of the camp which was located in a no-man’s land, Site 2, contained only a little rubble and scrubland beyond the checkpoints of a still-contested border. The vast expanse of land looked underused and desolate. Signposts around these sites indicated that minefields had been cleared, but we stuck to the paths. What the humanitarians gathered to do was therefore not to visit a site of memory, the scenography of emotion tied to a ‘lieu de mémoire’,^30^ a tourist spot populated by ruins or monuments, but really to create a communal experience.^31^ The small crowd gathered around a battery-operated megaphone, passed from one person to the next, which transferred to one volunteer after another the right to tell their story. They often began by stating: ‘Another personal story from me’.^32^

These accounts were often centred on experiences – such as the evacuation in 1985 from the bombed refugee camps. This evacuation became a march organised by humanitarians at a time when the Vietnamese and their allies of the Kampuchean Revolutionary Armed Forces sought to destroy what were effectively the base camps of resistance to their rule. The mass exodus took the form of a demonstration – to use Danielle Tartakowsky’s definition^33^ – an identity parade unifying helpers and refugees in a common plight and genuine danger.

Most accounts relied on anecdotes to convey unspeakable life-changing experiences. American veterans recalled their cultural shock – taking showers in the rain and watching children riding astride buffaloes as they drove around the crowded dusty roads. Privately the recollections were on culture shock: the buffaloes now replaced by ‘iron buffaloes’ (wheeled multipurposed engines), the poverty of the Thai countryside which required matching relief from humanitarians. There were light moments when people recalled heavy drinking and the parties of the International Committee of the Red Cross (ICRC), but these moments of levity were framed by a broader and intense empathy for a traumatised nation of genocide survivors.

The recollection of sociability is also a feature of refugee experiences of the camps, and it is one which lives online. Cambodian refugees obtained access to cameras and could picture their lives – NGOs were keen to address the lost image that haunted Rithy Panh’s documentary making and cinema since.^34^ The pre–Khmer Rouge music scene was revived by teenager bands, and the slide shows available today on YouTube sequence images of young and smiling people facing the camera set against a melancholy and nostalgic soundtrack of innocence lost.^35^ This political imagining of a pre-turmoil Cambodia is of course apolitical and ahistorical. The five years of the Khmer Republic, under the dictatorship of Marshal Lon Nol, a regime backed by the USA after Prince Sihanouk was deposed in 1970, were hardly the years of insouciant prosperity many films recall today.^36^ Yet the victimhood of Cambodians in camps was not in doubt – they were all deserving recipients of aid and humanitarians were set up in austere environments nearby.

The lack of emotional distance between helpers and beneficiaries was often commented on. Mary, a nurse who had moved from San Francisco to work in the camps, knew little of the history of the Indochinese and Vietnam wars and little of the Khmer Rouge. She was moved by images of distress without much understanding of politics, and relocated to Aranyaprathet to help. There she lived a life distant from her Californian background:

We lost electricity a lot – you could not have a conversation during the monsoon if you lived in a house with a tin roof … I liked that you were in charge … everybody was helping each other. I would sit in the comfort of my house and look out and see a boy riding a buffalo. Life was very slow, we worked very hard … we had a maid. In a typical house, kind of liked that … big lizards that lived in the house. Getting into the bathroom at night, the bathroom was outside of the house. There was a tub of water in which you dipped. One of the houses I lived in. There was a big lizard living there … it created real anxiety. I left in 1982. That was the end. I found a nice connection when I came back to San Francisco [working with refugees].

The combination of what Rajak and Stirrat describe as ‘parochial cosmopolitanism’ and nostalgia is a recurrent feature of many of these testimonials, but they also reveal a depth of lifelong transformational engagement which transcends scholarly condescending.^37^ Mary kept working with refugees as a nursing manager and developed healthcare work addressing post traumatic stress disorder (PTSD) among the traumatised resettled. PTSD, which only entered the psychiatric Diagnostic and Statistical Manual of Mental Disorders in 1980 (DSM-III), particularly seemed to affect the refugees of Cambodia and also their helpers.^38^ Mary came back to the USA but her consciousness was durably altered.

When I left I kind of wanted to stay here but the programme had closed down. My visa ran out and I went back to the US. … My heart was here. It had changed my view of the USA and the western world and opened up my eyes to what the world is like. … I could not take things for granted … I did [become more politically active]… working with our legislators to get the issue of the Khmer Rouge holding seat in UN. … It gave us a sense of purpose. I guess it was hard to have a sense of purpose over there … Where I worked I would hear clerical staff talking badly of refugee situation … there was that level of conflict and I would try to explain the situation. There were many people who knew what happened with Khmer Rouge, they were interested and were in awe. How my god how could you do that.^39^

The emotional legacy of experience was thus dual: the aid worker was transformed by a temporary experience over the long term – their *becoming* a humanitarian had an existential impact which would endure.^40^ The highly mediatised image of ‘white saviours’ rushing to the rescue of distant sufferings was projected on the humanitarians themselves precisely at a moment when they could least reflect on their own inadequacies.^41^ The valorisation of humanitarian work was a double-edged sword for veterans who struggled to reintegrate into civilian life. The white saviour narrative that framed the image of their work at home contrasted with their sense of powerlessness while attempting to meet impossible large needs. The interest Mary describes for her work in Cambodia was reflecting sustained political focus on the victims of communism throughout the 1980s and on the significance of the Cambodian border for the development of humanitarian NGOs.^42^ Many veterans of other contexts such as the Ogaden refugees in Somalia or Mozambiquan refugees in Malawi would not have experienced any kind of recognition then. Of course, Mary also contrasted her experience with the difficulties refugees faced on resettlement in the USA.^43^

## 2 The Camps as the Ideal World of Humanitarian Norm-Setting?

From a purely normative perspective, camps were sites of norm-setting and where the urge to quantify was redefined afresh.^44^ The Cambodian camps had initially been managed by United Nations Children’s Fund and the ICRC, with UNHCR taking over the management of Khao I Dang as a camp of resettlement; but in 1982, UNBRO was set up for ordering NGOs and coordinate their action.^45^ As the archives of Stéphane Rousseau and his autobiography reveal, these were spaces where protection acquired new and more expansive meanings.^46^ The need to sustain what became a prolonged emergency created normative desires – as Vincent Fauveau recalled in a recent interview. The need to control what medicine could be undertaken became the heart of guidelines which later became standardised across the humanitarian sector.

The doctors who arrived from France had very little training. MSF then did not ask for a humanitarian background. So we were receiving people who had no idea of the specificity of humanitarian medicine and the medical work we had to do over there.^47^

The archives of Ron Ockwell at the University of Manchester also show vividly the normative processes arising from camp experience and from the need to curtail the constant flow of ‘experts’ seeking an ‘austere environment’ experience.^48^ The camp tourism of short-lived humanitarian volunteers was framed by efforts to normalise aid, to shape a common list of drugs available for use – all listed according to a common definition – and to decide on what could be attempted and what could not. The debates on ‘adequate’ aid and on culturally sensitive responses were found in every domain of humanitarian intervention:^49^ from the shaping of crude but effective prostheses by what became known as Handicap International to the definition of common medical standards.^50^ Rudi Coninx, who later became senior policy advisor at the World Health Organisation, reflected on his humanitarian experience in Cambodia as the most significant period of his education in humanitarian medicine at a time when standards were a ‘thing’:

So you have the Italians and they have a list of medicines that they can’t live without. And the next team happens to be a Finnish team who have never heard about these medicines, and want a whole set of different. And so before you know, you have stocks of all kinds of different medicines. So the standards was sort of like the – it was, it was a bit of a thing at the time.^51^

The emergence of standards has since been integrated into a collective professional life account of learning and development. Another veteran present at the Aranyaprathet gathering, Margie Ferris, reflected on how she developed her specific approach to nutrition in the camps and from that experience contributed to wider agenda setting debates of the following decades: ‘I did some training on negotiating complex settings’; ‘I have been commenting on every aspect of Sphere, on nutrition’; ‘We did [shape] we had standards that we developed here with the World Food programme … it kind of grew and grew.’^52^

The experience of normalising large refugee camps which were constantly traversed by political and criminal activities was also to confront en masse issues of trauma, physical and moral, apathy and violence, including sexual violence.^53^ As Rithy Panh recalled from his documentary making as a refugee filming in a refugee camp:

[In Site 2] Everything came in from the outside, including rice, water (for sanitation), and so on. Without water, everything is nothing. The refugees’ lives were so difficult, depending totally on outside help. I listened to the refugees talk about why they came to this camp, the gunfire they had dodged while they were fleeing. For them, living in a camp was not easy. During the day, there was not as much danger, but at night, it was a different situation. Some women were raped. Some were abused and kidnapped. They needed to be protected. They were vulnerable and had nothing to rely on.^54^

There was then little in terms of processes to quantify and assist victims of sexual violence, yet the process of humanitarian protection began to integrate these factors, while sensitivity to differences of what women, children and adolescents experienced in the camps became the basis of humanitarian programmes of aid. While the resourcing of Cambodian refugees per capita constantly exceeded that of those in other settings, the camps were neither stable nor contained. Trade and exchanges extended through their porous borders. Rice, in particular, was often sold on the black market to fuel the movements resisting the Vietnamese invaders. The UN operations were often alleged to turn a blind eye to these appropriations of aid by ‘refugee warriors’.^55^

While one needs to further nuance and research in more depth the material culture which enabled epistemic communities of care to develop and refine a common ‘field of action’,^56^ most humanitarians who experienced the Thai border refugee camps concur in stating that this experience developed as normative processes and principles. Some then jump to the late 1990s to argue that the Sphere project, usually framed as a response to the collapse of standards in the refugee camps of Goma in 1994 in Zaire in the aftermath of the Rwanda genocide,^57^ owed its focus on professionalising norms to this golden age of humanitarian experience.

If the experience of humanitarian work became retroactively legitimate because of its transformative influence on the long-term history of humanitarian aid, some humanitarians recalled how they were attracted to it at a personal level. Jack Dunford, who later spent his working life helping manage the camps on the border of Myanmar, referred to a kind of transformative religious experience and described ‘Something mysterious – you may not know you were being called. I was not religious’. Dunford worked in Thailand then, and he became aware of another refugee situation emerging five years after the sudden influx of Cambodians: ‘Karen refugees came in 1984’; ‘It was a tiny side show (5 March 1984–4 months initially) there were only 9 or 10,000 refugees. They were more like hill villages … what happened here [the Cambodian camps] was formative [for the Karen]’.^58^

## 3 The Legitimacy of Humanitarian Experience

The legitimacy of this emotional call to humanitarian work in the camps, what distinguished ‘real’ humanitarians with a labour to perform, was based on possessing some fundamental skills deemed of relevance to the emergency. Some, like Susan and Patricia Walker, possessed linguistic skills since childhood;^59^ others could offer medical skills, although often not specialised ones. Jean-Baptiste Richardier, who later founded Handicap International, recalled how he became an obstetrician through camp practice, while he had only graduated from French medical school as a generalist.^60^ The founder of MSF Logistics and the organiser of much of its pharmacy, Jacques Pinel, recalled in 2017 the original disappointment of a disorganised experience of aid:

I was disappointed originally, good people but no real know how … I knew why it was a mess and what to do … I had an image of MSF as saviours of humanity – it was very disappointing … on TV it was like it existed but there was nothing behind them … People who had left everything at full speed. There were misunderstanding … I [told them] I will propose myself as go to man of the team.^61^

Some of the humanitarians who served the longer times at the border camps were defined by their openness to ‘Oriental mores and culture’.^62^ Jean-Pierre Hiegel, who developed humanitarian psychiatric work in Khao I Dang, was one of these humanitarian workers who embraced the local religious belief systems to complement their allopathic practices.^63^

What enabled being a humanitarian was the ability to function socially and culturally over a period of time in a context of shifting allegiances and working practices. Forty years later, veterans recollected the networks across nationalities and among a young cohort of idealistic internationalists redefining what aid might mean and who they might be through delivering it. For many this was a transformational experience and a political coming of age. As Norah Niland recalls: ‘Up to that point I saw myself as a development person dealing with structural inequalities … but when I found myself with the survivors of the Khmer Rouge in close camps and some of the camps run and managed by the Khmer Rouge … I was there for four years … the politics were cruel to put it mildly’.^64^ Others remained apolitical but concerned with the power plays at the heart of their own practices.

The memories of this happenchance era are often glamorised. For instance, Jack Dunford recalled introducing Nick White, with whom he played cricket in Bangkok, to the Karen camps. Nick White became a very significant actor in medical research for the university consortium of Oxford and Mahidol which led important work on Falciparum malaria and artemisinin combination therapy.^65^

The mingling of social experience – mind altering experience – and new sensorial awareness to the world’s inequalities is in large measure the root of the legitimacy of humanitarian work (looking inwards) and defining belonging to the community of humanitarians. This sense of belonging was always tinged with a sense of inadequacy and despair. In practice, despite some hubristic statements, humanitarian work could not meet all needs. Humanitarians justified being there ‘to cast a spotlight’ on need, as Peter Walker put it.^66^ Having been there, having seen things and experienced traumatic circumstances was the defining trait of belonging to the group. It was a shared experience of a sort that defined legitimacy in humanitarian circles and helped shape identity along the lines of the work of Alasdair Gordon Gibson’s focus on volunteering.^67^

Reflecting on this history was not simply an isolated event to which a historian was invited. Forty-five years after initial scoping missions in 1976, MSF left Cambodia at the end of its final mission in the country on the treatment of hepatitis C. The same historian had been present in this final mission. While MSF developed an ambitious programme of aid associating high tech screening using Genexpert machines and extremely effective drug therapies,^68^ the leadership of the mission in Cambodia wished to reflect on whether there was a space of a Cambodian MSF: a shared past of working with and for MSF in Cambodia. It set up a Facebook group, and the advocacy officer, a medical doctor from Myanmar, Dr Sue Myat Han, was tasked by the head of mission Mick Le Paih to organise an exhibition focusing on the history of MSF in Cambodia.

It is not a celebration, it is not good to say that having an NGO in a country for 40 years is not something to celebrate … we could originally say 40 years with Cambodian people … we do not use 40 anymore (2019). The original idea was to have an associated movement … [Mick] was touched by this talking with a doctor in Phnom Penh. She knew MSF well since she was young because her mother worked for MSF for ten years as a counsellor, translator and social worker. Now her mum retired from MSF but she is working for MSF. He wanted an associate movement across the provinces … this generation is not really aware of the [Khmer Rouge] problems … we want to motivate them, that is why we aimed at the medical and paramedical students … the message is that you are not the recipients all the time, but you can give.^69^

The brief was to be environmentally friendly and bring Cambodian perceptions of humanitarian work. Myat Han reconstituted the history of MSF in Cambodia through interviews with old hands in the movement and Cambodian employees of the past. The exhibition was based on photographs, and she explained her choice of iconography as being specifically emotional: ‘I decided according to my emotions’. Other parties in the committee commissioning the exhibition ended up moderating Myat Han’s most unsettling choices, but an emotional thread ran throughout the exhibition. The exhibition was organised in four sections: what MSF is; the pathway through the history of MSF; the Hepatitis mission; and the presence of MSF in the South Asian region. The exhibition was composed of interactive panels which linked to recordings of patients and testimonials of humanitarian workers. The aims of the exhibition were thus to reach out to the nurses and doctors who had worked for MSF. It also funded an exhibition which travelled from one nursing school to another in Kampot, Kampong Cham, Battambang, Stung Treng and Phnom Penh. Beyond reaching out to the professional public –in the hope of increasing the visibility of MSF in Cambodia – the exhibition attempted to establish a coherent image of what humanitarian aid had done in the country and how it rested on a universal volunteering ethos.

The exhibition ‘Connecting Hands’ claimed to elicit what being ‘an MSF’ and what humanitarianism might be. The exhibits were associated with the projection of four films recorded a few years beforehand on other settings which dramatised emergency work and recalled the access campaign. This unifying discourse was in marked contrast with the exhibition itself, which reflected the great diversity of interventions funded and resourced by MSF. The panels included treatments of tuberculosis, malaria, aid for sex workers in Poipet, hepatitis C, reinforcing the health system and so on. In many ways the exhibition illustrated best not the constancy of humanitarian work but its bittiness, its inconsistent presence, associated with its high technicality. Meanwhile the emotional bonds made visible in the choice of images and evoked in the interviews reinforced the symbolic significance of humanitarian moral labour. Looked at from the perspective of one of those who worked for MSF or would work for the organisation, the exhibition showed its history as a long but disjointed sequence of employment opportunities. Missions followed each other, but each panel appeared to be engaging with a specific problem Cambodia had and was facing in the domain of health, with limited obvious connections between them. As an exhibition it presented a kind of narrative non sequitur of missions following each other in the broader Cambodian space but without reference to the politics that had kept MSF out of the inside of the country until 1991. The 1976–91 period was one during which MSF engaged continuously and assiduously with Cambodians, but only in the camps that were then on the margins of the country. The work of MSF during that period was one entwinned with Cold War necessities.

The years that followed showed the great flexibility of notions of humanitarian work. The succession of ‘missions’ undertaken by MSF showed how an emergency medical organisation responded to, but also shaped, a number of narratives of emergencies. This engagement with local authorities justified, until 2021, its presence in the country. While the exhibition itself was clearly aimed at Cambodians and expressed the notion that it was Cambodians themselves who had shaped their humanitarian experience, it nevertheless failed to become the catalyst of a Cambodian MSF organisation. Without external resources MSF Cambodia closed down and its Facebook group, MSF Cambodia Asso, is now a dead site, the final pages of which are precisely images from the ‘Connecting Hands’ exhibition.^70^

## 4 Conclusion

Reminiscing or accounting to the Cambodian (medical) public what it is to be a humanitarian – including by showing long and largely irrelevant films – was part and parcel of an act of recollection around experiences. The self-narratives I recorded, in the bus or in a collective passing of the megaphone were literally the voicing of experience as ‘embodied practices, sensations, feelings, emotions and thoughts’.^71^ It was an attempt to find a common ground for a group who had had a share of humanitarian experience, as care givers or recipients and often participants in aid. But it was not necessarily a common experience. There were then many different temporalities which applied for these testimonies, and which conflated different camps which were often closed to each other. The workers and refugees in Khao I Dang lived in a different world from those of Site 2, the people who experienced the wilderness of unruly criminal camps or the latent conflictual threat of warrior refugees – all contributed to the shaping of a recognisable form of sociability around humanitarian work. But this sociability was rooted primarily in work and in developing shared norms and practices.

Defining identity through the performance of work done and shared aspirations – even if some challenged elements of this narrative – was meant to be a consensual act which reminds us of veteran experience-sharing in military units. The same desire to commemorate and build a monument to the victims of this humanitarian carceral universe jarred somewhat with the overall bonhomie of the events. The questions asked by the general whose soldiers once terrorised refugees were responded to frankly, unemotionally and in terms of realpolitik. CIA agents were outed in the bus jokingly – more discrete were the indications that a sullen figure was once Khmer Rouge. In comparison, the exhibition ‘Connecting Hands’ similarly brought voices from the past and some recognised themselves in the images. The exhibition was set out around the patients’ voices rather than the experience of work, and it justified humanitarian work through needs rather than shared work culture or recollections of performative international solidarity.^72^ The exhibition attempted to present humanitarian aid in Cambodia as a Cambodian story of local agency. Arguably the humanitarian experience of Cambodian workers within the humanitarian health system was a tale of employment and education – access to resources and ideas rather than an emotional attachment. The youth in uniforms who politely visited the exhibition in their Chinese-built surroundings showed some interest in humanitarian work but related to this history only as a field of employment. The history of Cambodia as the recipient of international aid likely echoed far less in what is now a fast-growing economy in receipt of massive foreign investment and on the brink of becoming a lower middle-income country. The affective community around humanitarian work was not immediately relatable. Arguably the sequence of employment of national staff did not necessarily translate into a visible and claimed identity. I witnessed this distancing when a nurse in a health centre in Rolpa, province of Battambang, told me, when I inquired, that she had worked for or with MSF in the 1990s. This was something the 2019 MSF workers did not know or realise. Sharing experience did not come spontaneously in Cambodia.

If it failed somewhat to reach out, emotionally charged accounts of past humanitarian experiences remain central to the cultural identity of what are now multi-billion dollar organisations. The ‘impossible dream’ has to find a narrative voice grounded in humility and personal storytelling.^73^ Thus, even in the richly appointed headquarters of an NGO exhibitions are required to bring together the history and experience of being a humanitarian worker. In 2021–22 MSF organised a T-shirt exhibition, which travelled the French provinces and was exhibited in film festivals from Clermont Ferrand to Douarnenez in Brittany, for instance,^74^ which commentators described as ‘strikingly original’. The exhibits evoked experiences through material remnants of corporate identity markers – the MSF T-shirt which signalled affiliation and status, and even a form of ‘protection’.^75^ The panels combined storytelling and personal memories, and the T-shirts proved to be a very variegated medium for reminiscing about formative experiences.^76^

**Figure 3:**
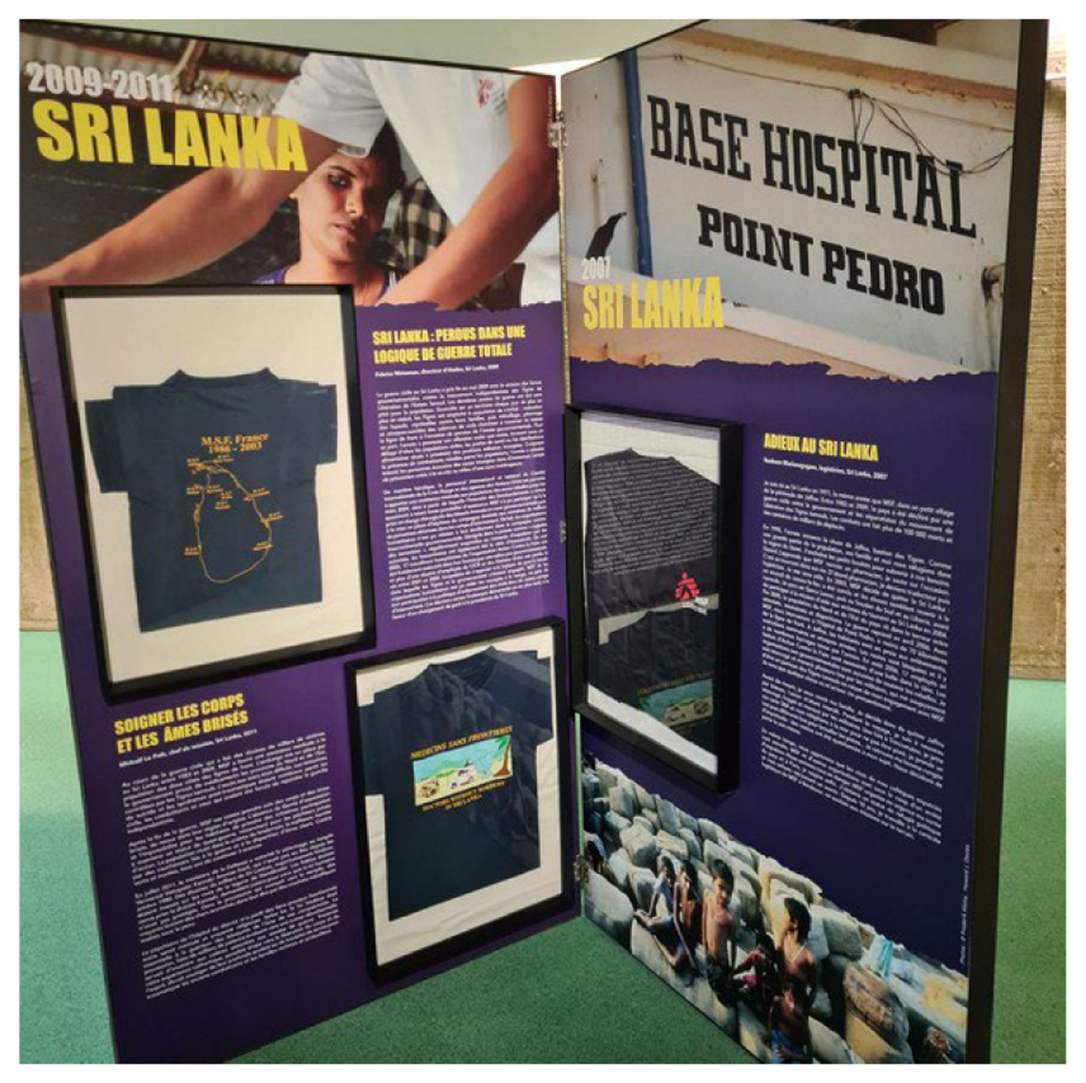
Sri Lanka 2009–11 panel, the MSF T-shirt exhibition in Paris, Spring 2022 With the Kind Authorisation of Nicolas Baudoin, Msf Paris

The panel pictured in figure 3, devoted to a particularly traumatising experience of humanitarian work in Sri Lanka, evoked well the difficulties of wartime humanitarian work. Throughout the exhibition, all panels associated T-shirts and a range of other garments with recollections of working for MSF. The intention was indeed to show how one ‘got’ the T-shirt. It revealed also how the T-shirts varied in their messaging and how experience was recalled by a range of humanitarian workers who belonged to MSF. They were MSF T-shirts who belonged to people who ‘were’ MSF. What the exhibition also revealed, implicitly, was how many of those garments had survived and how they had become treasured belongings. The moral labour they evoked was not only for others to witness – the performance of humanitarian work was deeply felt as a substantive part of humanitarians’ lives.^77^ Their affective appeal was commensurate to the profound experience they were associated to. Been there, got the T-shirt.

## Figures and Tables

**Figure 1:**
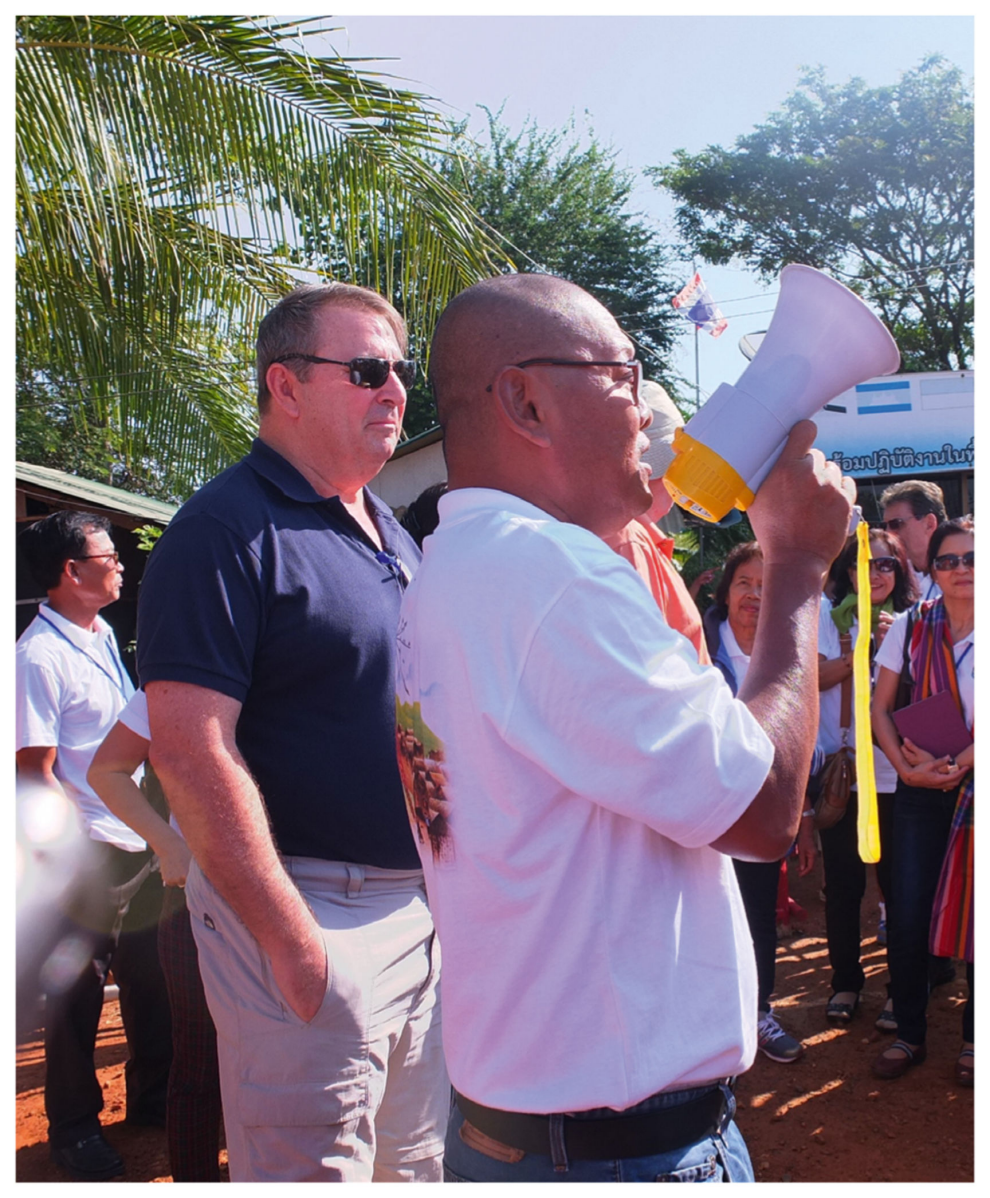
The megaphone in Khao I Dang, 10 January 2015 Photograph by the Author

**Figure 2:**
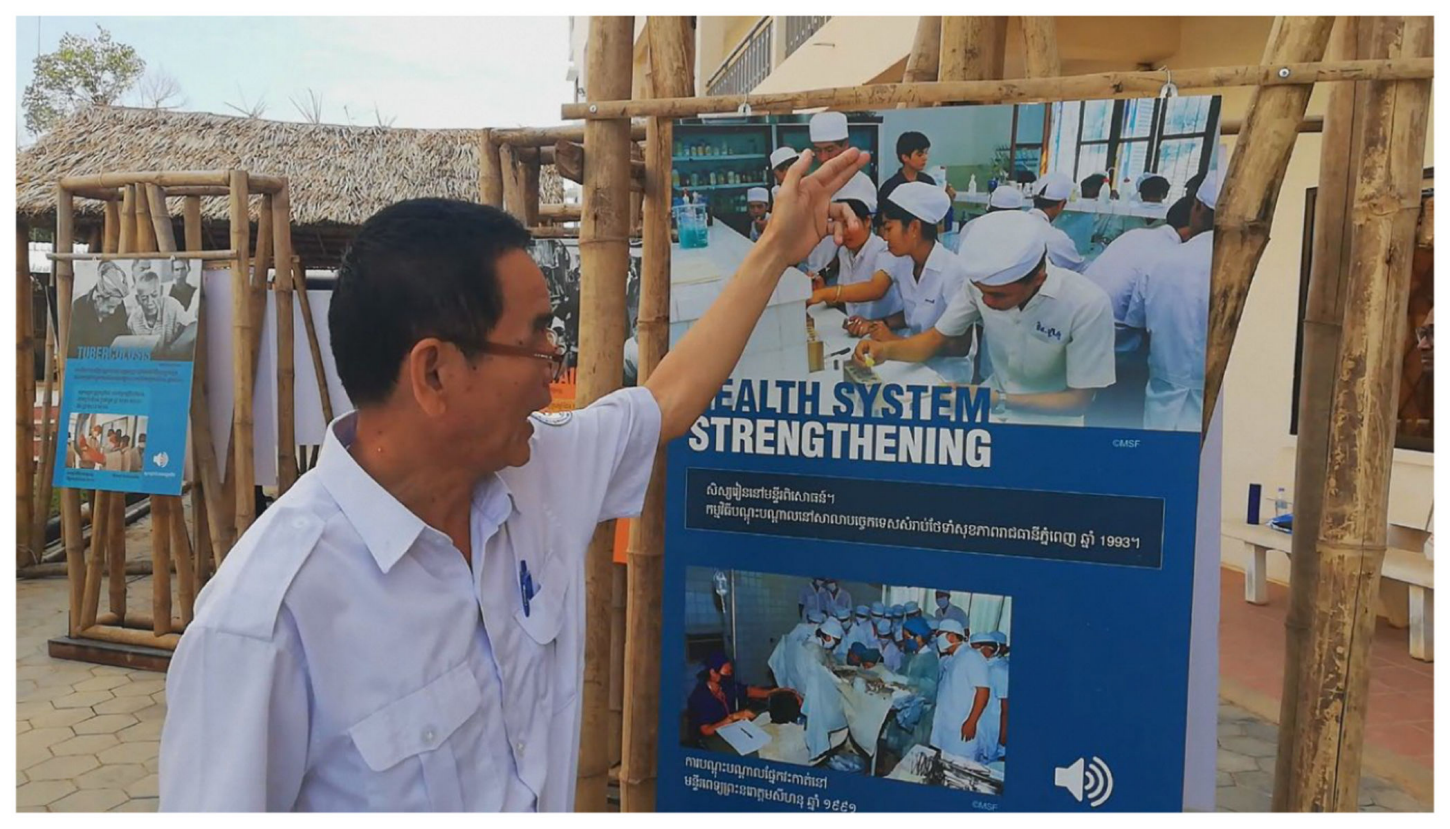
‘Connecting Hands’ in Battambang. A former MSF worker recognises himself on the display. With the Kind Authorisation of Sue Myat Han, Advocacy and Communication Manager Msf

## Footnotes

1 On the history of veterans and the meaning of the terms, there is a vast literature to which I am indebted. See Grace Huxford et al., ‘Writing Veterans’ History: A Conversation on the Twentieth Century,’ *War & Society* 38, no. 2 (2019): 115–38, for a recent summarising intervention.

2 The notion of humanitarian affective community has been deployed in International Relations scholarship: see, for instance, Amoz Hor, ‘The Everyday Emotional Lives of Aid Workers: How Humanitarian Anxiety Gets in the Way of Meaningful Local Participation,’ *International Theory* 14, no. 2 (2022): 358–87; Maja Povrzanović Frykman, ‘Sensitive Objects of Humanitarian Aid: Corporeal Memories and Affective Continuities,’ in *Sensitive Objects: Affects and Material Culture*, ed. Jonas Frykman and Maja Povrzanovic Frykman (Lund: Nordic Academic Press, 2016), 79–104; Juha Käpylä and Denis Kennedy. ‘Cruel to Care? Investigating the Governance of Compassion in the Humanitarian Imaginary,’ *International Theory* 6, no.2 (2014): 255–92 (258); and also, for a subtle exploration of this theme, see Peter Redfield, *Life in Crisis: The Ethical Journey of Doctors Without Borders* (Berkeley: University of California Press, 2013).

3 Rachel Coghlan, ‘Broaden the Narrative this World Humanitarian Day: Honour Those who Comfort the Dying,’ *The Lancet* 398, no. 10301 (2021): 659.

4 Michaël Neuman, ‘Dying for Humanitarian Ideas: Using Images and Statistics to Manufacture Humanitarian Martyrdom,’ Crash, 15 February 2017, https://www.msf-crash.org/en/publications/humanitarian-actors-and-practices/dying-humanitarian-ideas-using-images-and-statistics.

5 I draw here on Monique Scheer’s analysis of emotions as social practice: Monique Scheer, ‘Are Emotions a Kind of Practice (and Is That What Makes Them Have a History)? A Bourdieuan Approach to Understanding Emotion,’ *History and Theory*, 51, no. 2 (2012): 193–220.

6 Peter Leese, ‘The Limits of Trauma: Experience and Narrative in Europe c.1945,’ in *Trauma, Experience and Narrative in Europe after World War II*, ed. Ville Kivimäki and Peter Leese (London: Palgrave Macmillan, 2022), 3–26.

7 Anne-Meike Fechter, ‘Aid Work as Moral Labour,’ *Critique of Anthropology* 36, no. 3 (2016): 228–43.

8 This is a different affective work from that performed in the writing of memoirs since it is about sharing and partaking, but it nevertheless comforts gendering work other accounts deliver. See Róisín Read, ‘Embodying Difference: Reading Gender in Women’s Memoirs of Humanitarianism,’ in *The Politics of Peacebuilding in a Diverse World*, ed. Xavier Mathieu and Pol Bargués-Pedreny (London: Routledge, 2020), 18–36; and Bertrand Taithe, ‘Humanitarian Desire, Masculine Character and Heroics,’ in *Gendering Humanitarianism: Politics, Practice, and Empowerment during the Twentieth Century*, ed. Esther Möller, Johannes Paulmann, and Katharina Stornig (London: Palgrave, 2020), 35–60.

9 Bertrand Taithe, Mickaël le Paih, and Fabrice Weissman, ‘Historicising Humanitarian Action: Synchronicity in Historical Research and Archiving Humanitarian Missions,’ *Journal of Humanitarian Affairs*, 4, no. 2 (2022): 49–56.

10 See Bertrand Taithe, ‘The Cradle of the New Humanitarian System? International Work and European Volunteers at the Cambodian Border Camps, 1979–1993,’ *Contemporary European History* 25, no. 2 (2016): 335–58.

11 Kevin O’Sullivan, *The NGO Moment: the Globalisation of Compassion from Biafra to Live Aid* (Cambridge: Cambridge University Press, 2021).

12 See David Mosse, ed., *Adventures in Aidland: The Anthropology of Professionals in Inter national Development* (New York: Berghahn, 2011).

13 Laurence McFalls, ‘Benevolent Dictatorship: The Formal Logic of Humanitarian Government,’ in *Contemporary States of Emergency: The Politics of Military and Humanitarian Interventions*, ed. Didier Fassin (New York: Zone Books, 2010), 317–34.

14 The notion of system was emerging in the 1980s, and the Cambodian crisis was a prime example in arguing for coordinated response. Social Council, ‘Strengthening of the Coordination of Emergency Humanitarian Assistance of the United Nations’ (Resolution 2011/8, United Nations, 1991).

15 Mai’a K. Davis Cross, ‘Rethinking Epistemic Communities Twenty Years Later,’ *Review of International Studies* 39, no. 1 (2013): 137–60.

16 The Humanitarian Archive at the John Rylands Research Institute and Library at the University of Manchester contains many collections such as the papers of Ron Ockwell, Norah Niland, Antonio Donini and others which clearly document this process. ‘The Humanitarian Archive,’ John Rylands Research Institute and Library, n.d., accessed 21 October 2024, https://www.library.manchester.ac.uk/rylands/special-collections/subject-areas/humanitarian-archive/.

17 The results were the setting up of Active Learning Network for Accountability and Performance in humanitarian action (ALNAP); see Niels Dabelstein,. ‘Evaluating Humanitarian Action,’ *Schweizerisches Jahrbuch für Entwicklungspolitik* 18 (1999): 63–74; and John Borton, ‘Recent Trends in the International Relief System,’ *Disasters* 17, no. 3 (1993): 187–201.

18 See the filmed interview with Peter Walker in Sphere, ‘The Sphere Story II – Groundbreakers,’ YouTube, 1 July 2015, https://www.youtube.com/watch?v=cdl6jT7aaJ0.

19 Peter Walker and Susan Purdin, ‘Birthing Sphere,’ *Disasters* 28, no. 2 (2004): 100–11; Margie Buchanan-Smith, *How the Sphere Project Came into Being: A Case Study of PolicyMaking in the Humanitarian Aid Sector and the Relative Influence of Research* (London: Overseas Development Institute, 2003); Joël Glasman, *Humanitarianism and the Quantification of Human Needs: Minimal Humanity* (London: Routledge, 2019).

20 See Fechter, ‘Aid Work as Moral Labour,’ 231.

21 On a broader framing of humanitarian exhibitions, see Sébastien Farré, Jean-François Fayet, and Bertrand Taithe, eds, *L’Humanitaire s’exhibe – The Humanitarian Exhibition* (Geneva: Georg, 2022), https://www.georg.ch/l-humanitaire-s-exhibe.

22 Emily Bauman, ‘The Naïve Republic of Aid: Grassroot Exceptionalism in Humanitarian Memoir,’ in *Global Humanitarianism and Media Culture*, ed. Rachel Tavernor and Michael Lawrence (Manchester: Manchester University Press, 2019), 3–108; Bertrand Taithe and John Borton, ‘History, Memory and “Lessons Learnt” for Humanitarian Practitioners,’ *European Review of History: Revue européenne d’histoire* 23, no. 1–2 (2016): 210–24.

23 See on this Bertrand Taithe, ‘Horror, Abjection and Compassion: From Dunant to Compassion Fatigue,’ *New Formations* 62 (2007): 123–36; Bertrand Taithe, ‘Compassion Fatigue: the Changing Nature of Humanitarian Emotions,’ in *Emotional Bodies: Studies in the Historical Performativity of Emotionş* ed. Dolores Martin Moruno and Beatriz Pichel (Champaign: University of Illinois Press, 2019), 242–62; and Bertrand Taithe, ‘Empathies, soins et compassions: Les Émotions humanitaires,’ in *Histoires des émotions*, ed. Jean-Jacques Courtine, Alain Corbin and Georges Vigarello, vol. 3 (Paris: le Seuil, 2017), 364–81.

24 Bertrand Taithe and Mickael le Paih, ‘Making Hepatitis C history? Médecins sans Frontières, Hepatitis C and Humanitarian Medicine in Cambodia 2016–2021,’ *Social History of Medicine* (forthcoming).

25 Nat J. Colletta and Michelle L. Cullen. *Violent Conflict and the Transformation of Social Capital: Lessons from Cambodia, Rwanda, Guatemala, and Somalia* (New York: World Bank Publications, 2000).

26 William Shawcross, *The Quality of Mercy: Cambodia, Holocaust, and Modern Conscience* (New York: Simon & Schuster, 1984); Tony Jackson, *Just Waiting to Die* (Oxford: Oxfam Research and Evaluation Unit, 1987).

27 Oral history, field recording, 10 January 2015.

28 Arthur C. Helton, ‘Asylum and Refugee Protection in Thailand,’ *International Journal of Refugee Law* 1, no. 1 (1989): 20–47 (35–36); Bertrand Taithe, ‘Between the Border and a Hard Place: Negotiating Protection and Humanitarian Aid after the Genocide in Cambodia, 1979–1999,’ in *Human Rights and Humanitarianism: A World of Differences?*, ed. Michael N. Barnett (Cambridge: Cambridge University Press, 2020), 219–34.

29 Marita Eastmond and Joakim Öjendal, ‘Revisiting a “Repatriation Success”: The Case of Cambodia,’ in *The End of the Refugee Cycle? Refugee Repatriation and Reconstruction*, ed. Richard Black and Khalid Koser (New York: Berghahn, 1999), 38–55.

30 Dominique Chevalier and Isabelle Lefort. ‘Le Touriste, l’émotion et la mémoire douloureuse,’ *Carnets de géographes* 9 (2016), https://doi.org/10.4000/cdg.644.

31 Since then, a modest memorial industry has set up the first tracks for this emotional tourism. See Elizabeth Benedict, ‘My Experience There,’ Khao I Dang Today, n.d., accessed 21 October 2024, https://khaoidangtoday.com/my-experience-there/.

32 Khao I Dang, recording, DHM, Humanitarian Archive, University of Manchester; Khao I Dang speech 10.00–13.15 mins].

33 Danielle Tartakowsky, *Les Manifestations de rue en France: 1918–1968* (Paris: Éditions de la Sorbonne, 2021).

34 For example, Rithy Panh, *La Famille Tan* (La Sept [Arte], 1995); Rithy Panh, *L’Image man quante* (Les Acacias, 2013); see Leslie Barnes and Joseph Mai, eds, *The Cinema of Rithy Panh: Everything Has a Soul*, (New Brunswick, NJ: Rutgers University Press, 2021).

35 For instance see ‘khao i dang doung chet By Keo Sarath| Keo Sarath Song| Keo Sarath Mp3| Keo Sarath Old Song,’ YouTube, 20 October 2015, https://www.youtube.com/watch?v=PfzSmGCYln0, a collection of images compiled by Rainna Sang; Ray Bin’s slideshow ‘Khao I Dang Sakeo Refugee Camps,’ YouTube, 22 June 2013, https://www.youtube.com/watch?v=o4UlrMOAqWo; or the harrowing song ‘Khao I Dang by Chunn Vanna,’ YouTube, 14 July 2011, https://www.youtube.com/watch?v=NLFKcSv8k2E.

36 Bunkong Tuon, ‘Inaccuracy and Testimonial Literature: The Case of Loung Ung’s *First They Killed My Father: A Daughter of Cambodia Remembers*,’ *MultiEthnic Literature of the United States* 38, no. 3 (2013): 107–25; Trisnian Ifianti and Anita Kurnia Rahman. ‘Characterization Analysis of the Main Characters of “First They Killed My Father” Movie by Angelina Jolie,’ *Journey: Journal of English Language and Pedagogy* 3, no. 1 (2020): 19–25.

37 Dinah Rajak and Jock Stirrat, ‘Parochial Cosmopolitanism and the Power of Nostalgia,’ in *Adventures in Aidland*, ed. Mosse, 161–76.

38 Didier Fassin and Richard Retchman, *L’Empire du traumatisme. Enquête sur la condition de victime* (Paris: Flammarion, 2007); Eve Bernstein Carlson and Rhonda Rosser-Hogan, ‘Cross-Cultural Response to Trauma: A Study of Traumatic Experiences and Posttraumatic Symptoms in Cambodian Refugees,’ *Journal of Traumatic Stress* 7, no. 1 (1994): 43–58; James K Boehnlein et al., ‘A Ten-Year Treatment Outcome Study of Traumatized Cambodian Refugees,’ *The Journal of Nervous and Mental Disease* 192, no. 10 (2004): 658–63.

39 Interview with Mary, bus recording, 10 January 2015.

40 Here I am referring to the feminist ontology of becoming as used by Claire Malcolm, ‘Being and Becoming: Humanitarian Intervention and the Constructed Duty of Justice’ (PhD thesis, University of Cardiff, 2009), 2–5. Martin Jay, *Songs of Experience: Modern American and European Variations on a Universal theme* (Berkeley: University of California Press, 2005).

41 Themrise Khan, Kanakulya Dickson, and Maïka Sondarjee, eds, *White Saviorism in Inter national Development: Theories, Practices and Lived Experiences* (Quebec: Daraja Press, 2023).

42 Maria Cullen, ‘NGOs as National Political Actors during the Cold War: A Comparison of Médecins sans Frontières and Oxfam’s Humanitarian Programmes in the Global South, 1979–1988’ (PhD thesis, University of Galway, 2023).

43 Aihwa Ong, *Buddha is Hiding: Refugees, Citizenship, the New America* (Berkeley: University of California Press, 2003).

44 Joël Glasman and Brendan Lawson, ‘Ten Things we Know about Humanitarian Numbers,’ *Journal of Humanitarian Affairs* 5, no. 1 (2023): 1–10.

45 Taithe, ‘The Cradle of the New Humanitarian System?’.

46 Stéphane Rousseau, *Tranches de vie d’un expat de l’humanitaire* (Paris: L’Harmattan, 2020).

47 Interview with Vincent Fauveau, author of *Pour une médecine différente en milieu défavo risé* (Paris: MSF, 1981), 8 January 2024. Also see MSF Paris archive [MSFPA], Jacques Pinel papers, box 1, Débuts guides années 1979–1980.

48 Ron Ockwell, Papers, Humanitarian Archive, University of Manchester, ‘Ron Ockwell Papers – training etc. 1975–1980s.

49 On this broader question, see Heidi Morefield, *Developing to Scale: Technology and the Making of Global Health* (Chicago: University of Chicago Press, 2023).

50 Interview with Jean-Baptiste Richardier, 4 March 2024, DHM, Humanitarian Archive, University of Manchester.

51 Rudi Coninx, interview (with Courtney Stickland), 13 March 2024, RIAH, Humanitarian Archive, University of Manchester.

52 Interview with Margerie Ferris, 9 January 2015, DHM, Humanitarian Archive, University of Manchester.

53 Heidi Hetz, ‘The Concept of the ‘Good Refugee’ in Cambodian and Hazara Refugee Narratives and Self-Representation,’ *Journal of Refugee Studies* 35, no. 2 (2022): 874–92.

54 Y-Dang Troeung et al., ‘A Forum on Remembering Cambodian Border Camps, 40 Years Later,’ *Canadian Literature* 246 (2021): 142–59.

55 Kirsten McConnachie, ‘Rethinking the ‘Refugee Warrior’: The Karen National Union and Refugee Protection on the Thai – Burma border,’ *Journal of Human Rights Practice* 4, no. 1 (2012): 30–56; Courtland Robinson, ‘Refugee Warriors at the Thai-Cambodian Border,’ *Refugee Survey Quarterly* 19, no. 1 (2000): 23–37; Peter Gatrell, *The Making of the Modern Refugee* (Oxford: Oxford University Press, 2013).

56 To borrow a term from Fanny Chabrol and Jean-Paul Gaudillière, *Introduction à la santé globale*, Repères Histoire (Paris: La Découverte, 2023).

57 John, Eriksson et al., *The International Response to Conflict and Genocide: Lessons from the Rwanda Experience. Synthesis Report* (London: Joint Evaluation of Emergency Assistance to Rwanda, 1996).

58 Interview with Jack Dunford, Aranyaprathet, 9 January 2015.

59 The story of the American Refugee Committee is told in Biloine W. Young, *My Heart It Is So Delicious: Setting the Course for CrossCultural Health Care: the Story of the Center for International Health* (Minneapolis, MI: Afton Historical Society, 2007).

60 Interview with Richardier, Lyon, 4 March 2024.

61 Interview with Jacques Pinel, 2012, DHM, Humanitarian Archive, University of Manchester.

62 Humanitarians of the 1980s were not immune to orientalist tropes and they sought to engage with refugees’ belief systems and culture, with varying degrees of success. Charles Kemp, ‘Cambodian Refugee Health Care Beliefs and Practices,’ *Journal of Community Health Nursing* 2, no. 1 (1985): 41–52.

63 Colette Hiégel-Landrac and Jean-Pierre Hiégel, *Vivre et revivre au camp de Khao I Dang* (Paris: Fayard, 1996); Jan Ovesen and Ing-Britt Trankell, *Cambodians and Their Doctors: A Medical Anthropology of Colonial and Postcolonial Cambodia* (Copenhagen: Nias Press, 2010).

64 Interview with Norah Niland, Geneva, 13 June 2023, Humanitarian Archive, University of Manchester.

65 Interview with Dunford, 9 January 2015. The story of ACT treatment and medical humanitarians is to be found in Suna Balkan and Jean-François Corti, ‘Malaria: Introducing ACT from Asia to Africa,’ in *Medical Innovations in Humanitarian Situations*, ed. Jean-Hervé Bradol and Claudine Vidal, Crash, 2011, accessed 29 March 2024, https://msf-crash.org/en/medical-innovations-humanitarian-situations/8-malaria-introducing-act-asia-africa.

66 Interview with Peter Walker, 16 October 2024.

67 Alasdair Gordon-Gibson, *Humanitarians on the Frontier: Identity and Access along the Borders of Power* (Lanham, MD: Rowman & Littlefield, 2021).

68 See Taithe and Le Paih, ‘Making Hepatitis C History?’

69 Interview with Sue Myat Han, Krong Stung Treng, 25 November 2019.

70 ‘MSF Asso Cambodia,’ Facebook, accessed 29 March 2024, https://www.facebook.com/search/top?q=msf%20asso%20cambodia.

71 Rob Boddice and Mark Smith, *Emotion, Sense, Experience* (Cambridge: Cambridge University Press, 2020), 23.

72 Lilie Chouliaraki, ‘Post-humanitarianism: Solidarity as Lifestyle Politics,’ in *Routledge Handbook of Humanitarian Communication*, ed. Lilie Chouliaraki and Anne Vertergaard (London: Routledge, 2021), 421–35.

73 Renée C. Fox, *Doctors without Borders: Humanitarian Quests, Impossible Dreams of Médecins sans Frontières* (Baltimore, MD: Johns Hopkins University Press, 2014).

74 ‘Douarnenez. Médecins sans frontières raconte cinquante ans de lutte avec des t-shirts,’ Ouest France, 24 August 2022, https://www.ouest-france.fr/bretagne/douarnenez-29100/douarnenez-medecins-sans-frontieres-raconte-cinquante-ans-de-lutte-avec-des-t-shirts-da8923ce-22f7–11ed-8661–a88a5b97efaf; ‘MSF au Rendez-vous du Carnet de Voyage,’ Médecins sans Frontières, n.d., accessed 29 March 2024, https://www.msf.fr/evenements/msf-au-rendez-vous-du-carnet-de-voyage.

75 Myfanwy James, ‘“Who Can Sing the Song of MSF?”: The Politics of “Proximity” and Performing Humanitarianism in Eastern DRC,’ *Journal of Humanitarian Affairs* 2, no. 2 (2020): 31–39.

76 Renée Fox noted the importance of T-shirts in humanitarian missions. Fox, *Doctors without Borders*.

77 Didier Fassin, *Humanitarian Reason: A Moral History of the Present*, trans. Rachel Gomme (Berkeley: University of California Press, 2011).

